# Glucocorticoids modulate human brown adipose tissue thermogenesis *in vivo*

**DOI:** 10.1016/j.metabol.2017.01.024

**Published:** 2017-05

**Authors:** Hannah Scotney, Michael E. Symonds, James Law, Helen Budge, Don Sharkey, Konstantinos N. Manolopoulos

**Affiliations:** a Division of Child Health, Obstetrics & Gynaecology, School of Medicine, University of Nottingham, Nottingham, NG7 2UH, UK; b Nottingham Digestive Diseases Biomedical Research Unit, Queens Medical Centre Campus, Nottingham, NG7 2UH; c Institute of Metabolism and Systems Research, University of Birmingham, Birmingham, B15 2TT, UK; d Centre for Endocrinology, Diabetes and Metabolism, Birmingham Health Partners, Birmingham, B15 2TT, UK

**Keywords:** ^18^FDG-PET/CT, 18F-fluorodeoxyglucose-positron emission tomography/computed tomography, ATP, adenosine triphosphate, AUC, area under the curve, BAT, brown adipose tissue, CRF, clinical research facility, DXA, dual energy x-ray absorptiometry, HC, hydrocortisone, HOMA, homeostatic model assessment, ISO, isoprenaline, IT, infrared thermography, NEFA, non-esterified fatty acids, NIHR, National Institute for Health Research, NRES, National Research Ethics Service, REC, research ethics committee, tAUC, time-averaged area under the curve, T_REF_, reference point temperature, T_SCR_, supraclavicular temperature, UCP1, uncoupling protein 1, β-AR, beta adrenoceptor, Brown adipose tissue, Glucocorticoids, Humans, Infrared thermography, Beta adrenoceptor

## Abstract

**Introduction:**

Brown adipose tissue (BAT) is a thermogenic organ with substantial metabolic capacity and has important roles in the maintenance of body weight and metabolism. Regulation of BAT is primarily mediated through the β-adrenoceptor (β-AR) pathway. The *in vivo* endocrine regulation of this pathway in humans is unknown. The objective of our study was to assess the *in vivo* BAT temperature responses to acute glucocorticoid administration.

**Methods:**

We studied 8 healthy male volunteers, not pre-selected for BAT presence or activity and without prior BAT cold-activation, on two occasions, following an infusion with hydrocortisone (0.2 mg.kg^− 1^.min^− 1^ for 14 h) and saline, respectively. Infusions were given in a randomized double-blind order. They underwent assessment of supraclavicular BAT temperature using infrared thermography following a mixed meal, and during β-AR stimulation with isoprenaline (25 ng.kg fat-free mass^− 1^.min^− 1^ for 60 min) in the fasting state.

**Results:**

During hydrocortisone infusion, BAT temperature increased both under fasting basal conditions and during β-AR stimulation. We observed a BAT temperature threshold, which was not exceeded despite maximal β-AR activation. We conclude that BAT thermogenesis is present in humans under near-normal conditions. Glucocorticoids modulate BAT function, representing important physiological endocrine regulation of body temperature at times of acute stress.

## 1 Introduction

There is increasing evidence that brown adipose tissue (BAT) has important physiological roles beyond thermoregulation in newborn infants and rodents [1]. Adult humans have significant amounts of BAT [2] and, as a highly metabolic tissue with the capacity to oxidize both glucose and lipid, attention has turned to its involvement in the pathogenesis of obesity and the metabolic syndrome [3]. BAT is characterized by the presence of uncoupling protein (UCP) 1 which uncouples adenosine triphosphate (ATP) production by the mitochrondrial respiratory chain, allowing the dissipation of excess chemical energy as heat [4]. The principal factors regulating BAT function in healthy adults have yet to be fully established due, in part, to the technical limitations of assessing BAT function *in vivo*. The majority of studies in humans have used 18F-fluorodeoxyglucose-positron emission tomography/computed tomography (^18^FDG-PET/CT) as the gold standard to assess BAT activity, but this is constrained by exposure to ionizing radiation, the scanning protocols involved [5] and its unsuitability for live tracking of BAT activation especially after feeding. Systemic β-adrenoceptor (β-AR) activation promotes BAT activity in humans [6], but the role of other endocrine factors remains largerly unknown. The pre-partum elevation of cortisol is pivotal in the initiation of nonshivering BAT thermogenesis at birth [7], and glucocorticoids have recently been proposed as regulators of BAT activity in healthy adult females [8] and in individuals pre-selected for the presence of active BAT [9]. BAT has also been considered to contribute to dietary-induced thermogenesis [10], [11], although this concept remains controversial [12]. We, therefore, studied whether BAT is activated by feeding, or by an acute increase in cortisol under basal and β-AR stimulated conditions.

## 2 Materials and Methods

### 2.1 Subjects

Eight healthy male volunteers participated in this randomized, double-blind, placebo controlled study, conducted between January and March 2015. Individuals were recruited using print and electronic advertising and none was selected or screened on the basis of presence of any active BAT. All subjects underwent a medical evaluation during a screening visit to ensure they were healthy. No subject had any significant past medical history, smoked tobacco or took any regular medications that could affect the study's outcome measures.

### 2.2 Study Approval

The study was approved by the Edgbaston NRES Committee, UK (REC reference 14/WM/1085). All participants provided written informed consent.

### 2.3 Study Design

#### 2.3.1 Clinical Research Facility

All parts of this study were conducted in a temperature controlled room at the National Institute for Health Research (NIHR)/Wellcome Trust Clinical Research Facility (CRF) of the University of Birmingham at the Queen Elizabeth Hospital Birmingham, UK. Room temperature was held constant at 23–26 °C and was monitored using an ambient temperature probe.

#### 2.3.2 Anthropometric Measurements

Measurements were taken during the screening visit. Waist circumference was measured midway between the lower margin of the last palpable rib and the top of the iliac crest, and hip circumference at the level of the greater trochanters. Total and regional fat masses were measured by dual-energy x-ray absorptiometry (DXA). Visceral fat mass was estimated by DXA using a proprietary algorithm provided by the manufacturer [13]. Core temperature was measured with a tympanic thermometer.

#### 2.3.3 Study Visits

**Fig. 1:**
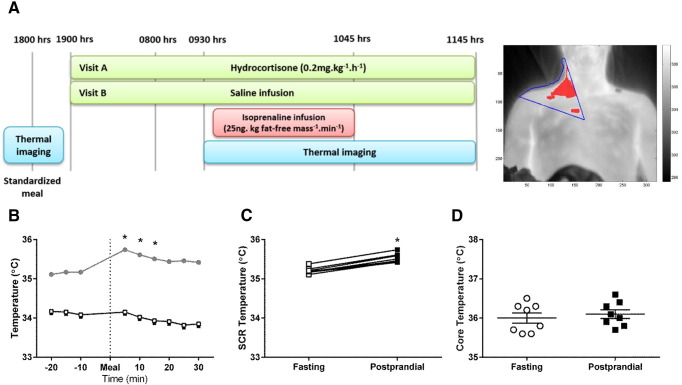
Study design and temperature responses to a meal. Each participant underwent the study twice, whereby BAT thermogenic activity was studied with infrared thermography before and after a standardized meal, followed by either a 14 h overnight constant hydrocortisone or normal saline (control) infusion. Infusions were given in a randomized, double-blind order and continued during and after β-adrenoceptor stimulation with isoprenaline (**A**). Mean changes in supraclavicular region (T_SCR_, gray circles) and non-adipose tissue reference (T_REF_, open squares) temperatures (dotted line indicates time of meal) (**B**), individual responses (fasting, open squares; postprandial, black squares) (**C**), and changes in core temperature (fasting, open circles; postprandial, black squares) (**D**) following the meal. *p < 0.05 compared to fasting baseline, n = 8.

Study visits were identical, except for the nature of overnight infusion, and were at least 2 weeks apart (Fig. 1A). Subjects were admitted to the CRF in the afternoon, and a cannula for infusion purposes was inserted into a right antecubital fossa vein. At 1800 h, they were served a standardized calorie-controlled meal (vegetable lasagne; total energy 2634 kJ; typical nutritional values per 100 g of product: 1.9 g fat, 12.2 g carbohydrates, 3.3 g protein, 1.5 g fiber), and then fasted until study completion the next day. BAT thermogenesis assessment was performed immediately before and after the meal, which was ingested within 20 min and was acompanied by tap water at room temperature. At 1900 h, a constant infusion of either hydrocortisone (HC, 0.2 mg.kg^− 1^.h^− 1^) or normal saline (control study visit) was started and given until study completion the following day. Infusions were administered in a double-blind, randomized fashion. At 2200 h, lights were switched off for night rest. In the morning, cannulations for blood sampling purposes were performed and, at 0900 h, the isoprenaline infusion protocol commenced. After baseline measurements for 45 min, a one-step infusion of isoprenaline (ISO, 25 ng.kg fat-free mass^− 1^.min^− 1^) was given for 60 min. BAT thermogenic activity was measured at baseline and throughout the infusion.

### 2.4 BAT Thermogenesis Assessment

An infrared thermography (IT) camera (FLIR E60 2.3 Megapixel Infrared Camera; FLIR Systems, Danderyd, Sweden) was used to acquire images of the anterior neck and upper chest region, which were sequentially analyzed and processed by an automated analysis program, as described previously [14]. Areas of interest for temperature analysis were the supraclavicular region (T_SCR_) representing BAT, and a non-adipose tissue reference point (T_REF_) on the chest, close to the xiphoid. In addition, during the periods of IT, two skin contact temperature sensors (iButton DS1922L, Maxim Integrated, Winnersh, UK) recording skin temperature every minute were taped within the supraclavicular fossa (main BAT site) and lateral to the umbilicus (white adipose tissue). For skin contact temperature measurements, data were collected every minute, and analysis was performed using 5-min averages. For meal meaurements, the mean of both study days was calculated. Fasting and pre-ISO baseline were defined as the average of time points − 15 to 0 min. Postprandial and peak post-ISO infusion periods were defined as time points 0 to 15 min and 40 to 50 min, respectively. For the duration of the study, participants were wearing a hospital gown, with their torso exposed for the duration of all measurements.

### 2.5 Analytical Methods

Blood samples were drawn into heparinized syringes, and plasma was prepared rapidly at 4 °C and immediately frozen at − 80 °C before analysis. Plasma glucose and NEFA concentrations were measured enzymatically using commercially available kits on an ILAB600 or ILAB650 clinical analyzer (Instrumentation Laboratory UK, Warrington, UK). Insulin and C-peptide were measured by ELISA (Invitron, Monmouth, UK) at a reference laboratory (Diabetes Research Unit Cymru, Swansea University, UK). Cortisol was measured by a colorimetric assay (R&D Systems, Abingdon, UK).

### 2.6 Calculations and Statistics

Indexes of β-cell function and insulin resistance were calculated according to the homeostatic model assessment (HOMA) method, whereby the mean of three consecutive plasma glucose and insulin postabsorptive measurements was used. Energy expenditure was calculated based on heart rate, age and weight as previously described [15]. Area under the curve (AUC) was calculated using the trapezoid rule and is presented as a time-averaged value (tAUC; AUC divided by the relevant time period). Comparisons between groups were analyzed using *t* test or non-parametric tests for data that were not normally distributed. A p < 0.05 was considered statistically significant. Based on previous studies using a similar integrative physiology design [16], the sample size was designed to have 85% power to detect a difference of 0.75 standard deviations at the 5% significance level for metabolic parameters. Data were analyzed using IBM Statistics for Windows v21 and GraphPad Prism for Windows v6.05. All data are presented as mean ± SEM, unless otherwise stated.

## 3 Results

### 3.1 Meal Ingestion Leads to Increased BAT Thermogenic Activity

Baseline anthropometric and metabolic characteristics of participants are shown in Table 1 and environmental temperature data for each individual study day are shown in **Supplemental Table 1**. There was no difference in outside or room temperature between study days. Following the mixed meal, postprandial T_SCR_ increased, whereas T_REF_ remained stable (Fig. 1B). All participants responded with an increase in BAT thermogenic activity (Fig. 1C), while core temperature did not change (Fig. 1D). Skin contact measurements showed a similar postprandial temperature increase of 0.39 ± 0.10 °C over supraclavicular BAT (p < 0.05 compared to fasting), whereas skin temperature over white adipose tissue remained the same.

### 3.2 Acute Hypercortisolemia Induces Peripheral Insulin Resistance and Increases Basal BAT Thermogenic Activity

**Fig. 2:**
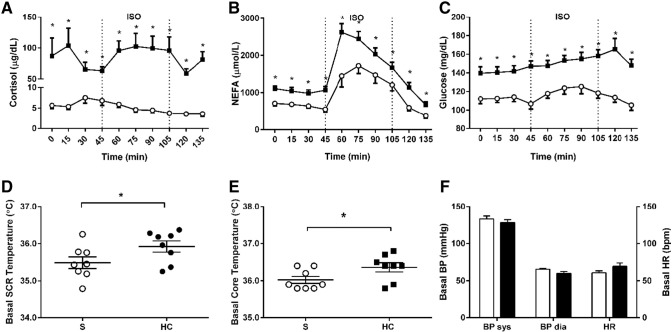
Metabolic and cardiovascular responses following saline infusion (S) or hydrocortisone infusion (HC) at baseline and during an isoprenaline infusion (ISO, dotted lines indicate infusion period). Plasma cortisol concentrations (saline, open circles; hydrocortisone, black squares) (**A**), non-esterified fatty acids (NEFA) (saline, open circles; hydrocortisone, black squares) (**B**), glucose (saline, open circles; hydrocortisone, black squares) (**C**), supraclavicular temperature (T_SCR_) (saline, open circles; hydrocortisone, black circles) (**D**) and core temperature at baseline (saline, open squares; hydrocortisone, black squares) (**E**). Systolic (sys) and diastolic (dia) blood pressure (BP) and heart rate (HR) at baseline (saline, open bars; hydrocortisone, black bars) (**F**). *p < 0.05 *vs.* control, n = 8.

Overnight HC infusion resulted in significantly increased plasma cortisol concentrations (Fig. 2A). From a metabolic perspective, basal plasma non-esterified fatty acids (NEFAs) were high due to fasting (Fig. 2B). HC increased basal NEFA and glucose (Fig. 2C), as well as insulin (basal insulin 30.4 ± 6.0 pmol/L *vs.* 55.2 ± 7.4 pmol/L, p = 0.025 control compared to hypercortisolemia) and C-peptide concentrations (basal C-peptide 0.25 ± 0.03 pmol/mL *vs.* 0.38 ± 0.04 pmol/mL, p = 0.001 control compared to hypercortisolemia). In line with this, HOMA indices of peripheral insulin resistance increased (HOMA IR index control 0.62 ± 0.11 *vs.* hypercortisolemia 1.11 ± 0.16, p = 0.016) (Table 2).

Acute hypercortisolemia increased T_SCR_ in the basal state (Fig. 2D). This was accompanied by an increase in basal core temperature (Fig. 2E), but we did not observe any effect on blood pressure or heart rate (Fig. 2F).

### 3.3 Acute β-AR Stimulation Increases BAT Thermogenic Activity During Control and Hypercortisolemia Conditions

From a metabolic perspective, ISO infusion significantly increased systemic NEFA concentrations, both under control and hypercortisolemia conditions (Fig. 2B). Despite the augmentation of basal systemic lipolysis by HC, the β-AR dependent rise in plasma NEFA was of similar magnitude compared to control conditions (Δ AUC 953 ± 155 *vs* 979 ± 175 μmol/L; p = 0.926 compared to control). Following the initial peak, there was a sharp decline in NEFA concentrations despite continuing ISO infusion. Control plasma glucose concentrations were unaffected by ISO, while the observed increase in concentrations during HC infusion is due to glucocorticoid-induced peripheral tissue insulin resistance (Fig. 2C). This is supported by the concomitant changes in insulin, C-peptide and HOMA indexes showing a decrease in glucose sensitivity despite a significant increase in insulin and C-peptide concentrations during ISO infusion (Table 1). Expectedly, non-selective β-AR stimulation with ISO increased heart rate and systolic blood pressure, responses not significantly affected by HC (Fig. 2F and Supplemental Fig. 1). Basal and ISO-induced BAT thermogenic activity measures did not show any significant correlation with BMI or measures of adipose tissue distribution (data not shown).

**Fig. 3:**
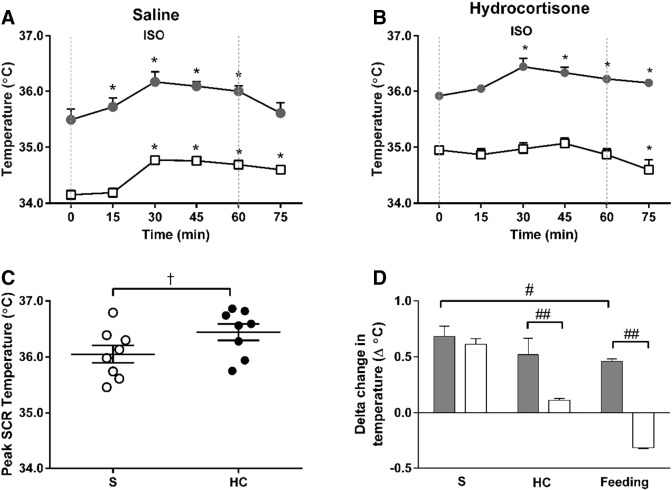
BAT thermogenic responses. Supraclavicular temperature (T_SCR_) and non-adipose tissue reference point (T_REF_) following saline (S) infusion (T_SCR_, gray circles; T_REF_ open squares) (**A**) or hydrocortisone (HC) infusion (T_SCR_, gray circles; T_REF_ open squares) (**B**) during and after isoprenaline stimulation (ISO, dotted lines indicate infusion period). Individual peak BAT temperatures during ISO (S, open circles; HC, black circles) (**C**). Change in temperature during ISO or following a standardized meal (T_SCR_, gray bars; T_REF_ open bars) (**D**). *p < 0.001 *vs.* basal, †p < 0.05 *vs.* saline, #p < 0.001 *vs.* saline, ##p < 0.001 *vs.* T_REF_, n = 8.

Adrenergic stimulation resulted in a highly localized increase in temperature within the supraclavicular region, representative of BAT thermogenic activity, both under control and hypercortisolemic conditions (Fig. 3). All study participants responded to ISO with an increase in BAT temperature (Supplemental Fig. 2). Under control conditions, ISO increased T_SCR_ by 0.7 °C, plateaued and then returned to baseline after the infusion, implying cessation of β-adrenergic-mediated BAT thermogenesis (Fig. 3A). These responses to ISO were similar during hypercortisolemia (Fig. 3B), whereby peak T_SCR_ was slightly higher (Fig. 3C). The ISO-induced T_SCR_ increase was greater than the physiological stimulus of diet-induced thermogenesis (Fig. 3D). Skin temperature showed similar results (Supplemental Fig. 3). Energy expenditure increased significantly during ISO (Supplemental Fig. 4). During hypercortisolemia, ISO-induced energy expenditure was closely correlated with basal T_SCR_ during control conditions (Pearson r = 0.742, p = 0.035) and peak T_SCR_ during HC (r = 0.870, p = 0.005). In response to ISO, peak core temperature was similar between control and hypercortisolemia conditions (Supplemental Fig. 5).

## 4 Discussion

Human supraclavicular BAT is characterized by the presence of thermogenically functional UCP1, with a respiratory capacity that substantially exceeds that of white fat [17]. Understanding the endocrine factors regulating BAT function is an important prerequisite before being able to utilize the metabolic capabilities of this tissue. In this study we sought to study BAT *in vivo* following exposure to a combination of physiological stimuli in order to determine the relative importance of diet and edocrine mediated effects.

BAT glucose uptake has been reported to be increased following a single carbohydrate-rich meal [18], although overfeeding for 24 h did not have any effect [19]. This has led to some controversy regarding the contribution of BAT to dietary-induced thermogenesis in humans. We sought to investigate this using a single standardized mixed meal, serving as a physiological stimulus. While we did not measure whole body energy expenditure, we observed selective temperature changes over the supraclavidular region only, immediately after the meal, suggesting direct BAT activation and not a thermic effect of food. Interestingly, from a mechanistic perspective, postprandial BAT activation would be characterized by both systemic cortisol secretion [20] and sympathetic β-AR stimulation [21], suggesting an acute maximal response following feeding.

Cortisol promotes important physiological maturation effects around the time of birth, including raised UCP1 abundance in adipose tissue [7], [22]. However, in adult rodents, glucocorticoids inhibit BAT [23] by interfering with adrenergic signaling [24], [25]. Human data are scarce with one study reporting dexamethasone-induced inhibition of UCP1 expression and metabolic rate in human brown adipocytes *in vitro* [26], and another reporting BAT activation following administration of the synthetic glucocorticoid prednisolone *in vivo* [9]. In our study, we chose hydrocortisone to model a physiological acute surge of cortisol, as seen during the perinatal period and at times of acute stress. We observed an increase of basal T_SCR_ during hypercortisolemia, supporting a physiological role for cortisol in BAT activation, as we did not observe any additive effects on blood pressure or heart rate. The duration of the infusion was chosen to allow for glucocorticoid-mediated genomic effects to take place [27]. While the achieved plasma cortisol concentrations were in excess of those typical for acute stress [28], it is important to note that tissue-responsiveness can be determined by tissue-specific glucocorticoid metabolism rather than absolute plasma concentrations [29]. Taken together our data indicate the positive relationship between cortisol and BAT temperature as previously indicated from a small study on healthy adult females [8].

β-AR stimulation induces BAT thermogenesis in humans [6], [30], although findings are inconsistent depending on the β-AR employed [31], [32]. We found a localized increase in supraclavicular temperature during ISO infusion both under control and hypercortisolemia conditions. This temperature change was temporally limited for the duration of the infusion, suggesting underlying BAT activation. T_SCR_ responses for all subjects increased within the first 5 min which is in accordance with acute cold exposure on BAT [14]. The observed T_SCR_ plateau is suggestive of a limit to BAT thermogenesis *in vivo*. The concomitant sharp decline in NEFA concentrations during the later stages of the infusion is consistent with β-AR desensitization due to maximal receptor stimulation [16]. The finding of a slightly higher peak T_SCR_ during hypercortisolemia suggests a minor synergistic effect between cortisol and β-AR stimulation. Interestingly, the two pathways are intrinsically connected as catecholamine synthesis is under glucocorticoid control [33].

Previous studies have shown that active BAT decreases with age and obesity, and its activation varies between sexes [14], [34]. We studied BAT activity in healthy males using IT to assess temperature changes in the supraclavicular region and a non-adipose tissue reference point. Supraclavicular skin temperature increases upon BAT activation [35], [36] and IT has been shown to measure changes in skin temperature overlying the main BAT depot in humans [14]. It has been confirmed as a reliable alternative for *in vivo* BAT activity assessment, correlating with ^18^FDG-PET/CT [9], [37], with the additional benefit of enabling real-time tracking of temperature changes. IT-derived BAT temperature measurements might be influenced by subcutaneous adipose tissue thickness [38], however, in our study participants were lean and we monitored dynamic temperature changes over time, as opposed to a single, static measurement. Adrenoceptor-induced vasodilation, both as a result of HC and ISO infusions, could increase skin blood flow and interfere with IT measurements. However, compared to T_SCR_, there were clear temporal differences in the change in T_REF_ which showed a later initial increase and a sustained increase post-infusion. Overall, we demonstrate a BAT-specific thermogenic and vasodilation response to both HC and ISO, clearly differentiated from non-BAT reference areas, confirming that the temperature responses we measured are confined to BAT.

Our findings support previous studies showing β-AR stimulation as a means of activating BAT in humans [6], [30], confirming IT as a sensitive, non-invasive method for the *in vivo* assessment of BAT function in humans under near-normal conditions [9], [14], [37]. This is particularly important when comparing our results with those studies using glucose tracer uptake as an index of BAT activity. Similar ISO doses did not show any significant BAT glucose tracer uptake, likely due to competition between the tracer and fatty acids from ISO-induced lipolysis combined with increased insulin resistance [32]. Given that BAT primarily utilizes fatty acids for heat generation [1], it is possible that ^18^FDG-PET/CT underestimates the amount of active BAT in humans. This limitation has led to the development of alternative BAT assessment methods, in addition to IT, either using different PET/CT tracers [39], or based on magnetic resonance imaging techniques [40]. We demonstrate that temperature changes in the supraclavicular area upon β-AR stimulation are indicative of localized BAT activity in a cohort of unselected young individuals, maintained at room temperature. This supports the prospect of harnessing BAT activity and the associated increase in energy expenditure as a potential treatment for metabolic diseases. We provide further evidence that in humans, in contrast to rodents, acute hypercortisolemia does not inhibit BAT function, but results in BAT activation [9]. Despite this, there is a threshold of activity that cannot be overcome even during maximal short-term β-AR stimulation.

Our study has some limitations by design, including the small size of our sample, although it is standard for a healthy volunteer study of this type. The acute infusion of hydrocortisone limits the conclusions we can draw in relation to states of chronic glucocorticoid excess that are associated with profound metabolic changes, *i.e.* Cushing's Syndrome. In addition, the concomitant induction of relative insulin resistance during hydrocortisone infusion might have obscured glucocorticoid-specific effects on BAT function, especially since there is a complex relationship between insulin-mediated glucose uptake and BAT perfusion and activity *in vivo* [41]. The strenghts of our study are the randomized double-blind design of the infusion protocol and that all measurements were carried out within a short period of time, reducing the confounding effect of variations seasonal temperature, and thus endogenous BAT activity. In addition, by using IT for the assessment of BAT thermogenic activity, we were able to perform live tracking of BAT function in response to experimental stimuli.

In conclusion, glucocorticoids modulate BAT thermogenesis and may represent an important physiological mechanism for maintaining human body temperature at times of acute stress. Our study suggests that transient stress could act to promote BAT function. This suggests that depending on the type and magnitude of stress BAT could be utilized to improve body weight regulation and metabolic homeostasis.

## Authors’ contributions

KNM and MES designed the study; HS and KNM conducted the experiments and analyzed the data with JL, HS, MES, JL, HB, DS and KNM wrote the manuscript.

## Funding

The study was funded by a Starter Grant for Clinical Lecturers of the Academy of Medical Sciences awarded to KNM; HS is supported by a Medical Research Council Doctoral Training Award.

## Conflict of interest

No conflicts of interest, financial or otherwise, are declared by the authors.

## Figures and Tables

**Table 1:** Baseline anthropometric and metabolic characteristics of participants, n = 8.

Characteristic	
Age (years)	20 (18–34)
Weight (kg)	75.0 (61.5–81.7)
BMI (kg/m^2^)	23.0 (20.6–24.2)
WHR	0.84 (0.78–0.9)
Trunk fat (kg)	7.7 (5.1–9.2)
Leg fat (kg)	5.5 (4.3–7.5)
Visceral fat (kg)	0.2 (0.1–0.4)
Systolic BP (mmHg)	124 (105–145)
Diastolic BP (mmHg)	72 (59–79)
Heart rate (bpm)	58 (53–62)
Fasting glucose (mg/dL)	113 (89–122)
Fasting insulin (pmol/L)	30.6 (14.4–68.5)
Fasting NEFA (μmol/L)	707 (450–873)
TSH (mIU/L)	1.30 (0.93–4.83)
FT4 (pmol/L)	17.7 (15.0–18.8)

Median and range shown. BMI, body mass index; WHR, waist-to-hip ratio; BP, blood pressure; TSH, thyroid stimulating hormone; FT4, free thyroxine.

**Table 2:** Comparison of insulin and C-peptide AUC, and homeostatic model assessment (HOMA) indexes during basal and isoprenaline-stimulated conditions, n = 8.

	Control		Cortisol		*P* value control *vs* cortisol		*P* value basal *vs* isoprenaline	
	Basal	ISO	Basal	ISO	Basal	ISO	Control	Cortisol
Insulin (pmol/L)	30.4 ± 6.0	100.4 ± 15.7	55.2 ± 7.4	206.7 ± 30.5	0.025	0.012	0.012	0.012
C-peptide (pmol/mL)	0.25 ± 0.03	0.47 ± 0.05	0.38 ± 0.04	0.90 ± 0.09	0.001	0.001	< 0.0001	< 0.0001


	Control	Cortisol	*P* value
Δ insulin basal to ISO (pmol/L)	70.0 ± 10.1	151.5 ± 28.9	0.012
Δ C-peptide basal to ISO (pmol/mL)	0.22 ± 0.03	0.52 ± 0.07	0.012
HOMA %B	42.2 ± 5.9	40.5 ± 4.5	0.731
HOMA %S	192.2 ± 29.5	105.9 ± 19.2	0.004
HOMA IR	0.62 ± 0.11	1.11 ± 0.16	0.016

Mean and SEM shown.

## Appendix A Supplementary data

Supplementary figures and tables Image 1
