# Supplementary material for: Glucocorticoids modulate human brown adipose tissue thermogenesis in vivo
Source: Metabolism. 2017 May;70:125–32. doi: 10.1016/j.metabol.2017.01.024 (PMC5395593; doi:10.1016/j.metabol.2017.01.024)
Supplement: Supplementary file 1 — Supplementary figures and tables [file mmc1.pdf]

Supplemental data

Supplemental Table 1: Environmental temperatures in relation to the time of year throughout which each study was conducted. Environmental outside temperature data obtained from the Meteorological office. Note overnight duration of infusion as outlined in main text.

Saline infusion					
Participant	Date of study	Environmental room temperature (°C)		Environmental outside temperature (°C)	
		Infusion start	Infusion end	Infusion start	Infusion end
1	13/01/2015	23.7	25.2	6	4
2	24/02/2015	22.8	25.8	6	5
3	11/02/2015	24.4	24.0	5	5
4	21/01/2015	24.2	24.6	4	4
5	22/01/2015	24.1	23.9	4	4
6	26/02/2015	24.2	23.2	5	5
7	26/03/2015	25.2	25.2	7	7
8	24/03/2015	24.1	24.6	7	7
Mean ± SEM		24.1 ± 0.2	24.6 ± 0.3	5.5 ± 0.4	5.1 ± 0.4

Hydrocortisone infusion					
Participant	Date of study	Environmental room temperature (°C)		Environmental outside temperature (°C)	
		Infusion start	Infusion end	Infusion start	Infusion end
1	10/02/2015	23.7	23.9	4	5
2	14/01/2015	24.0	25.3	4	5
3	15/01/2015	22.2	23.5	5	4
4	25/02/2015	25.5	26.2	5	5
5	12/02/2015	24.0	24.8	5	4
6	25/03/2015	25.2	23.6	7	7
7	11/03/2015	25.1	25.2	6	6
8	12/03/2015	25.3	23.6	6	5
Mean ± SEM		24.4 ± 0.4	24.5 ± 0.4	5.3 ± 0.4	5.1 ± 1.0

Supplemental Figure 1

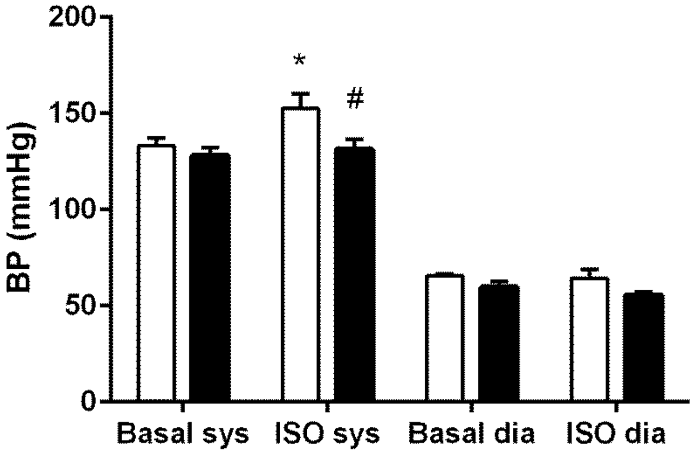

Systolic (sys) and diastolic (dia) blood pressure before (basal) and after 50 min of isoprenaline infusion (ISO), under control conditions (open bars) and following an overnight hydrocortisone infusion (black bars). * p<0.05 compared to basal control, # p=<0.05 compared to basal hydrocortisone, n=8

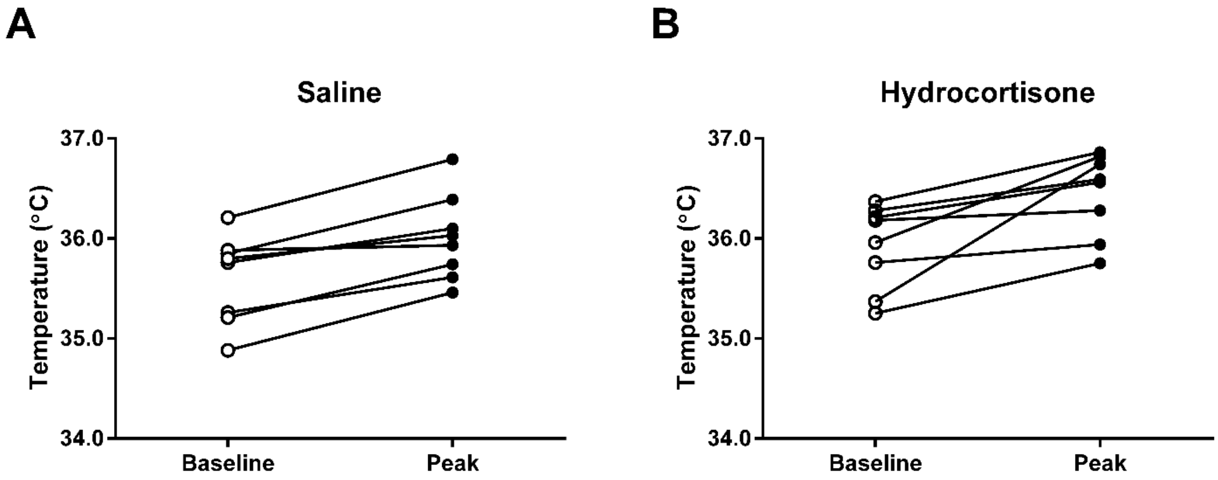

Supplemental Figure 2

Individual BAT temperature responses from baseline (open circles) to peak (black circles) following saline infusion **(A)** or hydrocortisone infusion **(B)**, n=8

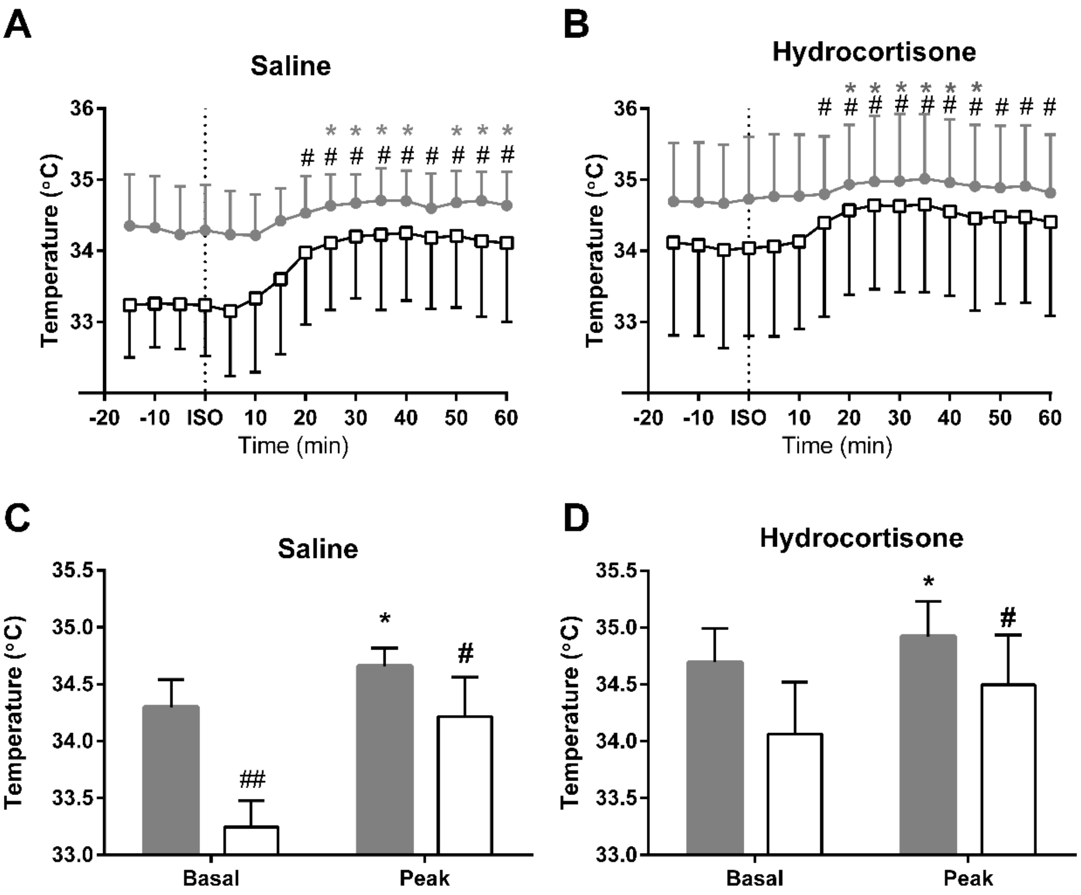

Supplemental Figure 3

Brown (BAT) and white adipose tissue (WAT) thermogenic responses to isoprenaline (ISO) using skin contact temperature sensors placed over the supraclavicular BAT region (grey circles and bars) or over an area of abdominal WAT (open squares and bars) under control conditions (**A**) and following an overnight hydrocortisone infusion **(B)**. Dotted line indicates start of ISO infusion. Mean temperatures at baseline and at peak during ISO (**C**, control and **D**, hydrocortisone). * $p<0.05$ peak/post-ISO BAT temperaure compared to basal BAT, # $p<0.05$ peak/post-ISO WAT temperature compared to basal WAT, ## $p<0.05$ compared to basal BAT, n=8

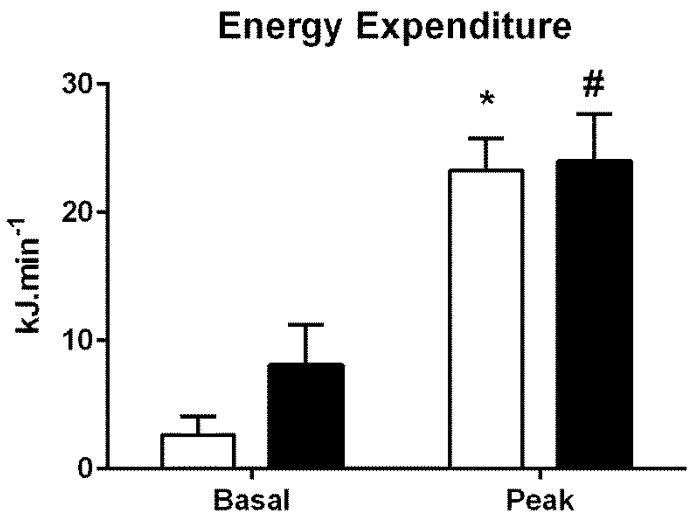

Supplemental Figure 4

Whole body energy expenditure under control conditions (open bars) and following an overnight hydrocortisone infusion (black bars) during basal conditions and after 50 min of isoprenaline infusion (peak). * $p<0.05$ compared to basal control, # p=<0.05 compared to basal hydrocortisone, $n=8$

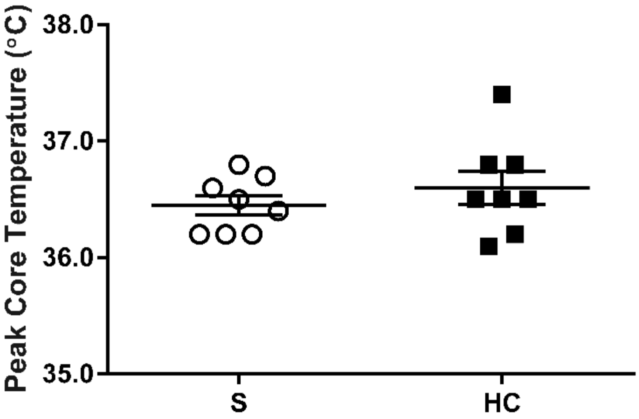

Supplemental Figure 5

Individual peak core temperature responses to isoprenaline infusion following saline infusion (S, open circles) or hydrocortisone infusion (HC, black squares), n=8
